# Individual factors predicted to influence outcome in group CBT for psychosis (CBTp) and related therapies

**DOI:** 10.3389/fpsyg.2015.01563

**Published:** 2015-10-28

**Authors:** Mahesh Menon, Devon R. Andersen, Lena C. Quilty, Todd S. Woodward

**Affiliations:** ^1^Department of Psychiatry, University of British ColumbiaVancouver, BC, Canada; ^2^Vancouver Coastal HealthVancouver, BC, Canada; ^3^BC Mental Health and Addiction Research InstituteVancouver, BC, Canada; ^4^Department of Psychology, University of SaskatchewanSaskatoon, SK, Canada; ^5^Campbell Family Mental Health Research Institute, Centre for Addiction and Mental HealthToronto, ON, Canada; ^6^Department of Psychiatry, University of TorontoToronto, ON, Canada

**Keywords:** psychotherapy, psychosis, cognitive behavior therapy, metacognitive training, MCT, CBT, psychotherapy outcome

## Introduction

Cognitive behavioral therapy for psychosis (CBTp) and CBT based interventions, such as Metacognitive Training for psychosis (MCT), have been found to be effective in symptom reduction, relapse prevention and, increased quality of life (Moritz et al., 2014). A number of reviews (Tarrier et al., 2002) and meta-analyses (Wykes et al., 2008) have confirmed that group CBT (including MCT) is effective for psychosis (Lecomte et al., 2012), but there is significant heterogeneity in outcome (Zimmermann et al., 2005), with some patients able to gain significantly from participating in psychotherapy, whereas others gain minimally. Given the limited resources available for psychotherapy in psychosis, an important endeavor is the identification of factors that may optimize the allocation of those resources (Zimmermann et al., 2005; Freeman, 2011). While there is a growing body of literature on factors influencing outcome in psychotherapy (Luborsky et al., 1971), that has examined patient, therapist and treatment factors, there is a relative paucity of research focussed on outcomes in group psychotherapy for psychosis. In this opinion paper, we highlight a number of patient factors (including aspects of symptomatology, cognition and personality) that might play a role in outcome in group psychotherapy for psychosis. These factors are based on research of psychotherapeutic outcomes (in psychosis and other disorders), with additional observations based on our clinical experience.

### Symptom related factors

#### Paranoia, lack of insight and their effects on treatment alliance

There is a large body of literature that has focussed on the non-specific factors associated with outcomes in other psychiatric disorders, and synthesizing it is beyond the scope of the current paper. The bulk of this research has looked at the importance of the therapeutic alliance (Martin et al., 2000), as perceived by the therapist and the client, and suggests that client perceptions of alliance are better predictors of outcome (Krupnick et al., 1996). Crucial to the alliance is the client's belief that they can trust the therapist, and feel understood by them (Frank and Gunderson, 1990). In the context of groups, an additional factor is the ability to trust the other group members. These critical components are negatively impacted by paranoia, and therapists working with clients with psychosis need to grapple with therapeutic ruptures and to avoid being integrated into the client's delusional framework.

CBT based interventions also require ways to discuss specifics of the delusional beliefs (such as the nature of evidence used to maintain the belief), as well as finding shared goals, even when clients have poor insight into their symptoms. This can be a delicate process, with a lot of potential for damaging the alliance. Goldsmith et al. (2015) found that the duration of therapy was associated with symptomatic improvement in those with good treatment alliance, but was actually detrimental for those clients with poor treatment alliance.

Skilled therapists find creative solutions to negotiate these processes. For some clients, particularly those with paranoia, relatively poor insight and an unwillingness to discuss the specifics of their delusional system, a more indirect approach (such as the one provided by MCT) (Kumar et al., 2015) may provide a more fruitful avenue of engagement (Menon et al., 2015). This is because MCT involves a group discussion of cognitive biases commonly associated with psychosis, and opportunities to examine the impacts of these biases in session, using non-delusion specific, and thus less threatening, material (Woodward et al., 2014).

#### Grandiose delusions

In general, one could argue that changing beliefs is difficult, due to the anxiety caused by any challenge to one's belief structure. However, there may be more motivation to challenge beliefs (such as persecutory delusions) associated with negative self-evaluations and anxiety (Bentall et al., 2001). In contrast, grandiose beliefs may be particularly intransigent to therapy as they may serve an ego protective function (Smith et al., 2006; Knowles et al., 2011). This may be particularly true when there is little associated distress, even if the belief has other negative effects on the client's relationships and life. Recent research (Garety et al., 2013) has suggested that patients with grandiose delusions may show even more pronounced cognitive biases (such as the jumping to conclusions bias) than those with persecutory (but non-grandiose) delusions. The goals of therapy may therefore need to be modified with this subgroup, with the initial focus being on minimizing the impact of their beliefs on their relationships. Unfortunately, these individuals also tend to be less receptive to challenges from peers. We therefore hypothesize that these individuals may benefit from individual psychotherapies (including MCT+), rather than group approaches.

It may also be worth exploring whether group therapy is more efficacious in individuals with similar types of delusions or dissimilar beliefs. The former provides a better milieu for common goal development, but could lead to inadvertent reinforcement of beliefs by group members, while the latter allows for members with differing beliefs to help each other in exploring disconfirmatory evidence.

#### Mood and anxiety

Depression, anxiety disorders and PTSD (Brady et al., 2003; Shevlin et al., 2008) are commonly comorbid with psychosis (Kessler et al., 2005). In socializing to the CBT model, we find that patients are more willing to explore the impact of their thoughts and behaviors on their depressive and non-psychotic anxiety symptoms. Thus, mood and “mood awareness” as targets for treatment may be initially less threatening than the potentially more threatening targeting of positive symptoms. Many participants identify with challenges and attribution biases related to depression and negative symptoms, while those associated with the positive symptoms are less frequently endorsed. Furthermore, addressing these difficulties can lead to an improved quality of life, as well as improved self-esteem and an increased sense of self-efficacy that can, in turn, build resilience and the capacity to challenge beliefs about hallucinations and targeting delusions.

### Neurocognitive deficits and illness duration

Neurocognitive deficits have been widely recognized as being a core feature of schizophrenia (Heinrichs and Zakzanis, 1998). Intervention studies for psychosis (both psychotherapeutic/CBTp and cognitive remediation) often involve exclusion criteria for individuals with learning disabilities or lower premorbid or current IQ (Garety et al., 2008; Freeman et al., 2014). In our groups, we find that patients may be able to comprehend core concepts, but may have difficulty retaining and understanding how these apply to their own symptoms or daily life. Nonetheless, recent research has suggested that neurocognitive functioning does not predict treatment retention (Baker et al., 2011) or treatment response in psychotherapy for psychotic disorders (Lincoln et al., 2014), and instead suggests that duration of illness is more closely associated with outcome, which may be indirectly related to cognition.

Although therapy might be helpful irrespective of the duration of the illness, it has repeatedly been noted that individuals might have maximal gains with early intervention (Lieberman et al., 2013). They may be more willing to challenge or change their beliefs given that these are not longstanding beliefs and patterns of thinking/behavior. Such individuals may tend to have more stable relationships and have not had the attrition in their social networks that occur over long duration of illness, and may be better able to engage in the social processes necessary to test alternative hypotheses.

### Social cognition

A growing body of research has suggested that social cognition might mediate the relationship between neurocognition and functional outcome in individuals with schizophrenia (Schmidt et al., 2011), thus indicating therapies that target and/or improve social cognition, such as social cognitive skills training (SCIT) (Horan et al., 2009; Roberts and Penn, 2009) and cognitive behavior social skills training (CBSST) (Granholm et al., 2007) may serve to improve functioning (Schmidt et al., 2011).

While MCT and CBT are distinctly different than social skills training, there is significant emphasis on improving social cognition through both psychoeducation (e.g., education regarding limitations in social perception, theory of mind) (Balzan et al., 2015), as well as exercises that encourage social interaction (during and outside group), and testing one's own abilities in these domains (Moritz et al., 2013). Although it may be hard to see overall differences between groups in head-to-head studies, we recommend studies comparing active interventions such as MCT and CBSST, to understand who might benefit more from social skills focussed interventions. Component analyses may provide another avenue by which to evaluate the utility of interventions across patient subtypes.

### Stigma

Stigma around psychosis and schizophrenia (both external and internalized) are common in our patients. In our experience, patient comfort with acknowledging their illness varies considerably. While patients may have the awareness of their symptoms and insight into how these symptoms manifest, they may be less comfortable discussing this in a group setting. We have noted that patients may take several sessions to endorse or engage in the therapeutic process. A true benefit of the group setting, and particularly a group with rolling intake, is that individuals can observe and attend to others' discussion of symptoms and self-chosen labels in order to gain comfort with terminology that they might not use in their everyday. Comparing approaches of a fixed entry vs. a rolling entry to groups and its impact on cohesion and stigma are areas that need to be examined in future studies.

### Personality factors

Psychotic disorders are associated with general personality pathology (Schultze-Lutter et al., 2014), as well as high neuroticism, and low openness, agreeableness, extraversion, and conscientiousness (Camisa et al., 2005). Personality traits such as psychoticism or schizotypy in particular have been highlighted as vulnerability factors for psychosis and potentially useful endophenotypes (Chmielewski et al., 2014). Yet, the prognostic or clinical utility of schizotypal personality features within treatment contexts has yet to be established.

Similar to neurocognitive factors, patient personality, particularly interpersonal features, have been linked to service utilization and clinical outcomes in patients with psychotic disorders. For example, high agreeableness and poor therapeutic alliance have been linked with poor treatment engagement and response (Lecomte et al., 2008). Other research has highlighted the impact of insight and attitudes related to recovery (such as optimism) (Lecomte et al., 2015) and stigma on alliance (Kvrgic et al., 2013) and outcome (Owen et al., 2015). In a group context, agreeableness, optimism and cohesion are particularly linked with positive outcomes (Lecomte et al., 2015).

### Participant selection in studies

We hypothesize that a part of the reason for the reduced effect sizes seen in randomized control trials (RCTs) lies in the characteristics of the participants who participate in research studies, such as personality (Kushner et al., 2009) and their levels of intrinsic motivation (Choi and Medalia, 2010). Some of our participants, when queried about their reasons for participating, pointed to extrinsic motivators (e.g., paid assessments, approval by their families etc.). In contrast, other participants gave reasons congruent with intrinsic motivation, even requesting to participate in additional groups and sessions despite no additional payment. Previous studies (Pelletier et al., 1997; Zuroff et al., 2007) of psychotherapy outcome in depression, and schizophrenia (Medalia and Richardson, 2005) found that intrinsically-motivated individuals generally make greater gains in psychosocial and cognitive remediation programs. Thus, we speculate that psychotherapy groups running without the financial incentives of the research assessment may have a greater proportion of participants with intrinsic motivation, and thus, better outcomes. Future studies may wish to explicitly examine reasons for participating in RCTs of psychotherapy for psychosis, using measures such as the Client Motivation for Therapy scale (Pelletier et al., 1997) and whether these are associated with outcome.

## Conclusions

Although this paper has focussed on group interventions, the majority of the research in this field has focussed on factors influencing outcome in individual therapy.

We have highlighted patient symptom, cognitive, and personality factors associated with therapeutic alliance and outcome (in individual and group therapy settings), which we hope will be examined in detail in future studies. It is important to acknowledge that for some of the factors we have highlighted, the effect size differences between groups might be small (e.g., comparing CBT to CBSST). This leads to a research challenge as many studies may be underpowered to address issues related to combinations of individual difference variables. Yet, the potential of this line of inquiry to reduce the personal and societal impact of psychosis is substantial, and has motivated similar initiatives in other disorders (Oquendo et al., 2014).

We suggest that this body of research can lead us down two distinct pathways. The first involves prospective studies to evaluate whether therapeutic benefits can be optimized by better identifying individuals who might benefit from therapy for psychosis in addition to traditional treatment. The second, more inclusive strategy, might involve examining these issues in large samples, perhaps by naturalistic study designs, or by creating treatment consortia to allow for pooled data, to look at cumulative benefits (e.g., of explicitly targeting attitudes related to recovery and stigma earlier in treatments), as well as comparisons of different active interventions. These approaches may allow us to directly compare multiple subgroups of participants, to see if these variations in treatment improve who might not otherwise benefit from “standard” treatment interventions.

## Funding

This work was partially supported by an Operating Grant from the Canadian Institutes of Health Research awarded to TSW and MM and a NARSAD Young Investigator Award to MM.

### Conflict of interest statement

The authors declare that the research was conducted in the absence of any commercial or financial relationships that could be construed as a potential conflict of interest.
